# Baseline D-Dimer Predicts Subsequent Venous Thromboembolism in 23,505 Patients with One of 11 Solid Malignancies: An 11-Year Retrospective Cohort Study

**DOI:** 10.3390/cancers18203307

**Published:** 2026-10-14

**Authors:** Yoshihiro Tanaka, Yuta Sato, Yuji Hatanaka, Ryoma Yokoi, Takeshi Horaguchi, Masahiro Fukada, Ryuichi Asai, Itaru Yasufuku, Yu Jesse Tajima, Nobuhisa Matsuhashi

**Affiliations:** Department of Gastroenterological Surgery Pediatric Surgery, Graduate School of Medicine, Gifu University, Gifu 501-1194, Japan

**Keywords:** cancer-associated thrombosis, D-dimer, venous thromboembolism, risk prediction

## Abstract

**Background/Objectives**: Venous thromboembolism (VTE) is a major complication of cancer. The predictive performance of baseline D-dimer and factors influencing its levels among patients with cancer remain unclear. We aimed to identify predictors of subsequent VTE development and evaluate the cancer-specific predictive performance of baseline D-dimer. **Methods**: This retrospective study included 23,505 patients with any of 11 malignancies who underwent baseline D-dimer measurement at initial diagnosis at Gifu University Hospital between January 2015 and December 2025. Patients with pre-existing VTE were excluded. Predictors were identified using logistic regression analyses. Cancer-specific predictive performance was evaluated using receiver operating characteristic analyses, with optimal cutoff values determined by the Youden index. Factors associated with baseline D-dimer were also assessed. **Results**: The median follow-up was 45.3 months (30.4–59.1 months), during which VTE occurred in 784 (3.3%) patients. Higher baseline D-dimer levels and distant metastasis were independent predictors of subsequent VTE. Cancer type was significantly associated with VTE overall, and breast cancer was associated with lower odds than esophageal cancer. D-dimer performance varied among cancer types, with optimal cutoff values of 0.55–2.35 μg/mL. Older age and higher platelet counts were independently associated with elevated baseline D-dimer levels, whereas a higher cholesterol level was inversely associated. **Conclusions**: Baseline D-dimer level is an independent predictor of subsequent VTE in patients with cancer. Its predictive performance and exploratory thresholds vary among cancers, suggesting that cancer type should be considered when interpreting baseline D-dimer levels. External validation is required before these cancer-specific thresholds can be applied clinically.

## Simple Summary

Venous thromboembolism (VTE) is one of the most serious complications in patients with cancer, and it is associated with high morbidity and mortality. The identification of high-risk patients before VTE develops is essential for timely prevention and management. In this large retrospective study that included 23,505 patients with multiple cancer types, we investigated whether baseline D-dimer levels at the time of cancer diagnosis predict subsequent VTE development. Baseline D-dimer level was an independent predictor of future VTE in the overall cohort. However, its predictive accuracy and optimal cutoff values varied considerably among different cancer types. These findings indicate that a single universal D-dimer cutoff value may not be appropriate for all patients with cancer. Instead, cancer-specific D-dimer thresholds may improve VTE risk stratification, enable earlier identification of high-risk patients, and inform future studies evaluating individualized VTE prevention strategies.

## 1. Introduction

Venous thromboembolism (VTE), including deep vein thrombosis (DVT) and pulmonary embolism (PTE), is one of the most common and clinically significant complications in patients with cancer [1,2,3,4]. Cancer-associated thrombosis contributes substantially to morbidity, mortality, interruption of anticancer therapy, and healthcare utilization [3,4,5].

The development of VTE in patients with cancer is multifactorial and involves interactions between tumor biology, host factors, treatment-related factors, and activation of the coagulation system [6,7,8,9,10]. Tumor cells promote thrombogenesis through tissue factor expression, procoagulant microparticle release, platelet activation, and inflammatory signaling pathways [11,12,13,14,15,16]. Consequently, biomarkers reflecting coagulation activation may provide useful information for identifying patients at increased risk of future thromboembolic events.

D-dimer is a degradation product of cross-linked fibrin, and it is widely used as a biomarker of coagulation and fibrinolytic activity [17]. Elevated D-dimer levels are associated with increased risks of VTE, disease progression, and mortality in patients with cancer [18,19,20,21]. However, previous studies have largely evaluated mixed cancer populations, and little is known about whether the predictive performance of D-dimer levels differs according to cancer type.

The Khorana score is one of the most widely used risk assessment models for VTE in patients with cancer [21]. However, this score was originally developed for outpatients undergoing chemotherapy and estimates VTE risk based on the primary cancer site, body mass index (BMI), and hematological parameters, including leukocyte, hemoglobin, and platelet counts. In the perioperative setting, a prior study involving patients undergoing abdominal surgery reported that a preoperative D-dimer level of 3.0 μg/mL was associated with an increased risk of perioperative DVT [22]. Additionally, in the prospective Cancer–VTE Registry, patients with a D-dimer level of 1.2 μg/mL or lower at their initial visit were excluded from VTE screening [23].

Thus, varying D-dimer cutoff values have been applied depending on the clinical setting and the study population. However, to the best of our knowledge, no large-scale study has evaluated the association between baseline D-dimer levels at the time of cancer diagnosis and the subsequent development of VTE during the course of various cancer treatments across multiple cancer types.

The prospective Cancer–VTE Registry included six types of solid malignancies and followed patients for 1 year. In the present study, we expanded the study population to 11 types of solid malignancies and used longitudinal electronic medical record data to evaluate subsequent VTE development over a longer follow-up period.

Therefore, this retrospective study aimed to identify predictors of subsequent VTE development and evaluate the cancer-specific predictive performance of baseline D-dimer levels across 11 solid malignancies.

In addition, we sought to determine the optimal D-dimer cutoff values for individual cancer types using receiver operating characteristic (ROC) analyses.

## 2. Materials and Methods

### 2.1. Study Population

This retrospective study included patients with one of 11 malignancies—esophageal cancer, gastric cancer, hepatobiliary and pancreatic cancer, colorectal cancer, breast cancer, lung cancer, urologic cancer, gynecologic cancer, head and neck cancer, malignant brain tumor, and bone and soft tissue sarcoma—who first presented to our hospital between January 2015 and December 2025. In cases of multiple synchronous cancers, the cancer site with the most advanced disease was used for classification. Consecutive patients with one of these 11 malignancies who underwent baseline D-dimer measurements at the initial visit were eligible for inclusion. Patients with preexisting VTE diagnosed at the initial visit were excluded. Patient data were extracted from the institution-wide electronic medical record system using CLISTA! (https://www.meiz.co.jp/clista/ accessed on 1 April 2026) an electronic medical record data extraction system at Gifu University Hospital. The database covers patients across all clinical departments; therefore, the study cohort was not restricted to patients treated in the Department of Gastroenterological Surgery and Pediatric Surgery. Patients with the 11 prespecified malignancies during the study period were systematically identified from this hospital-wide database and screened according to the predefined inclusion and exclusion criteria.

The CLISTA! data extraction for this study was designed to collect detailed clinical and laboratory variables from patients with a baseline D-dimer measurement; therefore, detailed study variables were not extracted for patients without a baseline D-dimer measurement. Follow-up was defined as the period from the initial cancer diagnosis to the last available clinical encounter or death, whichever occurred first. For patients who developed VTE, the occurrence of VTE during the follow-up period was recorded. Patients without documented VTE were censored at the date of their last available clinical encounter or death. The data lock date was 30 June 2026. Because this was a retrospective study based on routinely collected electronic medical records, follow-up duration varied according to the date of initial presentation and the availability of subsequent clinical records.

### 2.2. Clinical Variables

Baseline demographic, clinical, and laboratory data obtained at the initial visit were collected from medical records. Variables included age, sex, body mass index, D-dimer level, white blood cell count (WBC), hemoglobin, platelet count, total protein, albumin, total cholesterol, triglycerides, C-reactive protein (CRP), hypertension, diabetes mellitus, current smoking, hepatic dysfunction, renal dysfunction, autoimmune disease, active infection, distant metastasis, and cancer type. According to the National Cancer Institute Common Terminology Criteria for Adverse Events (CTCAE) version 5.0 [24], grade ≥2 elevations in liver function parameters (aspartate aminotransferase, alanine aminotransferase, or bilirubin) and serum creatinine were considered indicative of hepatic dysfunction and renal dysfunction, respectively. Active infection was defined as the presence of a clinically diagnosed infectious disease documented during the initial consultation, including pneumonia, urinary tract infection, infectious enterocolitis, and other infectious conditions. D-dimer concentrations were measured using a latex immunoturbidimetric assay (LIAS AUTO D-Dimer Neo; Sysmex Corporation, Kobe, Japan) on an automated blood coagulation analyzer (CN-6500; Sysmex Corporation). D-dimer values were reported in μg/mL D-dimer units (DDU), with a reference range of ≤1.0 μg/mL. The laboratory reporting range was 0.5–3200 μg/mL. The assay reagent, analytical platform, unit convention, and reference range remained unchanged throughout the study period.

### 2.3. Outcome

The primary outcome was the subsequent development of VTE during follow-up. VTE was defined as PTE or symptomatic proximal lower-extremity DVT confirmed by contrast-enhanced CT. DVT events were restricted to clinically relevant, symptomatic proximal lower-extremity DVT identified from the electronic medical record database. PTE included both symptomatic events and incidental PTE detected on CT performed for cancer staging, treatment response assessment, or other clinical indications. The median follow-up duration for the overall cohort was 45.3 months (interquartile range (IQR), 30.4–59.1 months). Median follow-up duration varied across cancer types: 43.2 months (IQR, 13.4–61.1) for esophageal cancer, 60.1 months (IQR, 44.9–71.1) for gastric cancer, 32.1 months (IQR, 22.4–41.1) for hepatobiliary and pancreatic cancer, 52.9 months (IQR, 44.9–71.1) for colorectal cancer, 70.1 months (IQR, 55.0–70.3) for breast cancer, 33.1 months (IQR, 24.7–42.9) for lung cancer, 43.4 months (IQR, 24.9–68.1) for urologic cancer, 55.3 months (IQR, 34.9–67.1) for gynecologic cancer, 32.4 months (IQR, 22.9–50.1) for head and neck cancer, 39.5 months (IQR, 24.4–54.6) for malignant brain tumor, and 29.1 months (IQR, 14.9–43.9) for bone and soft tissue sarcoma.

### 2.4. Statistical Analysis

Patient characteristics were summarized according to cancer type. Continuous variables were presented as medians (interquartile ranges) and categorical variables as numbers (percentages). Univariable logistic regression analyses were performed to identify predictors of subsequent VTE development. Variables with a *p*-value < 0.05 in the univariable analyses were entered into a multivariable logistic regression model to identify independent predictors of VTE. Odds ratios (ORs) and 95% confidence intervals (CIs) were calculated. The multivariable logistic regression analysis was performed using complete cases. Multicollinearity among variables included in the multivariable logistic regression model was assessed using variance inflation factors (VIF). Model discrimination was evaluated using the area under the receiver operating characteristic curve (AUC) calculated from the predicted probabilities of the multivariable model. Calibration was assessed using the Hosmer–Lemeshow goodness-of-fit test and by comparing mean predicted probabilities with observed VTE incidence across deciles of predicted risk.

Cancer-specific ROC analyses were performed to evaluate the discriminative performance of baseline D-dimer levels for subsequent VTE. For cancer types with statistically significant ROC results, optimal D-dimer cutoff values were determined by maximizing the Youden index (sensitivity + specificity − 1). Sensitivity and specificity were presented with exact 95% confidence intervals calculated using the Clopper–Pearson method. Positive predictive value (PPV) and negative predictive value (NPV) were also calculated for each cancer-specific cutoff. To quantify uncertainty in the estimated cutoff values, 1000 bootstrap samples were generated separately for each cancer type with a statistically significant ROC result by sampling patients with replacement. The optimal cutoff was re-estimated by maximizing the Youden index within each bootstrap sample, and percentile-based 95% confidence intervals for the cutoff values were obtained from the resulting bootstrap distributions. Internal validation of the cancer-specific cutoff performance was additionally performed using bootstrap optimism correction. For each bootstrap sample, the Youden-optimal cutoff was determined and its performance was evaluated both in the bootstrap sample and in the original dataset. Optimism was defined as the mean difference in the Youden index between these two evaluations across 1000 bootstrap resamples. The optimism-corrected Youden index was calculated by subtracting the estimated optimism from the apparent Youden index in the original dataset. To account for differences in follow-up duration, an additional Cox proportional-hazard regression analysis was performed using the same covariates as the multivariable logistic regression model. Time to event was defined as the interval from initial presentation to VTE diagnosis or, in patients without VTE, to the last clinical encounter or death. The proportional-hazard assumption for D-dimer was assessed using its interaction with logarithm (ln) (time). Kaplan–Meier curves and the log-rank test were used to compare time to subsequent VTE among cancer types.

To better understand the clinical factors associated with baseline D-dimer levels, exploratory analyses were performed to identify factors associated with D-dimer levels. Univariable linear regression analyses were initially conducted, followed by multivariable linear regression analysis, including variables with a *p*-value < 0.05 in the univariable analyses. D-dimer levels were natural ln transformed before regression analyses because of their skewed distribution. Regression coefficients (B), 95% CIs, and *p*-values were calculated.

Missing data were minimal in the final analytic cohort. Missing values were present for WBC (*n* = 2), hemoglobin (*n* = 2), total protein (*n* = 20), albumin (*n* = 14), total cholesterol (*n* = 9), triglycerides (*n* = 11), smoking status (*n* = 4), and distant metastasis (*n* = 1); all other variables used in the analyses had complete data. Missingness was <0.1% for every variable. No imputation was performed. Cases with missing values for variables required for a given analysis were excluded from that analysis.

All statistical analyses were performed using SPSS Statistics software (Version 27, IBM Corp., Armonk, NY, USA). Statistical significance was defined as a two-sided *p*-value <0.05.

This study was reported in accordance with the Strengthening the Reporting of Observational Studies in Epidemiology (STROBE) statement.

## 3. Results

### 3.1. Patient Characteristics

Of the 24,376 patients who visited the hospital during the study period, 23,505 met the eligibility criteria and were included in the final analysis.

The baseline patient characteristics are summarized in Table 1. The cohort comprised patients with one of 11 different malignancies, including esophageal cancer (*n* = 1146), gastric cancer (*n* = 3455), hepatobiliary and pancreatic cancer (*n* = 2425), colorectal cancer (*n* = 4208), breast cancer (*n* = 2363), lung cancer (*n* = 4492), urologic cancer (*n* = 1808), gynecologic cancer (*n* = 1462), head and neck cancer (*n* = 796), malignant brain tumor (*n* = 432), and bone and soft tissue sarcoma (*n* = 918). Compared with patients without VTE, those who developed VTE had significantly higher baseline D-dimer levels and a higher prevalence of distant metastasis. The distribution of cancer types also differed significantly according to VTE status (Table 2).

### 3.2. Predictors of Subsequent VTE Development

**Figure 1:**
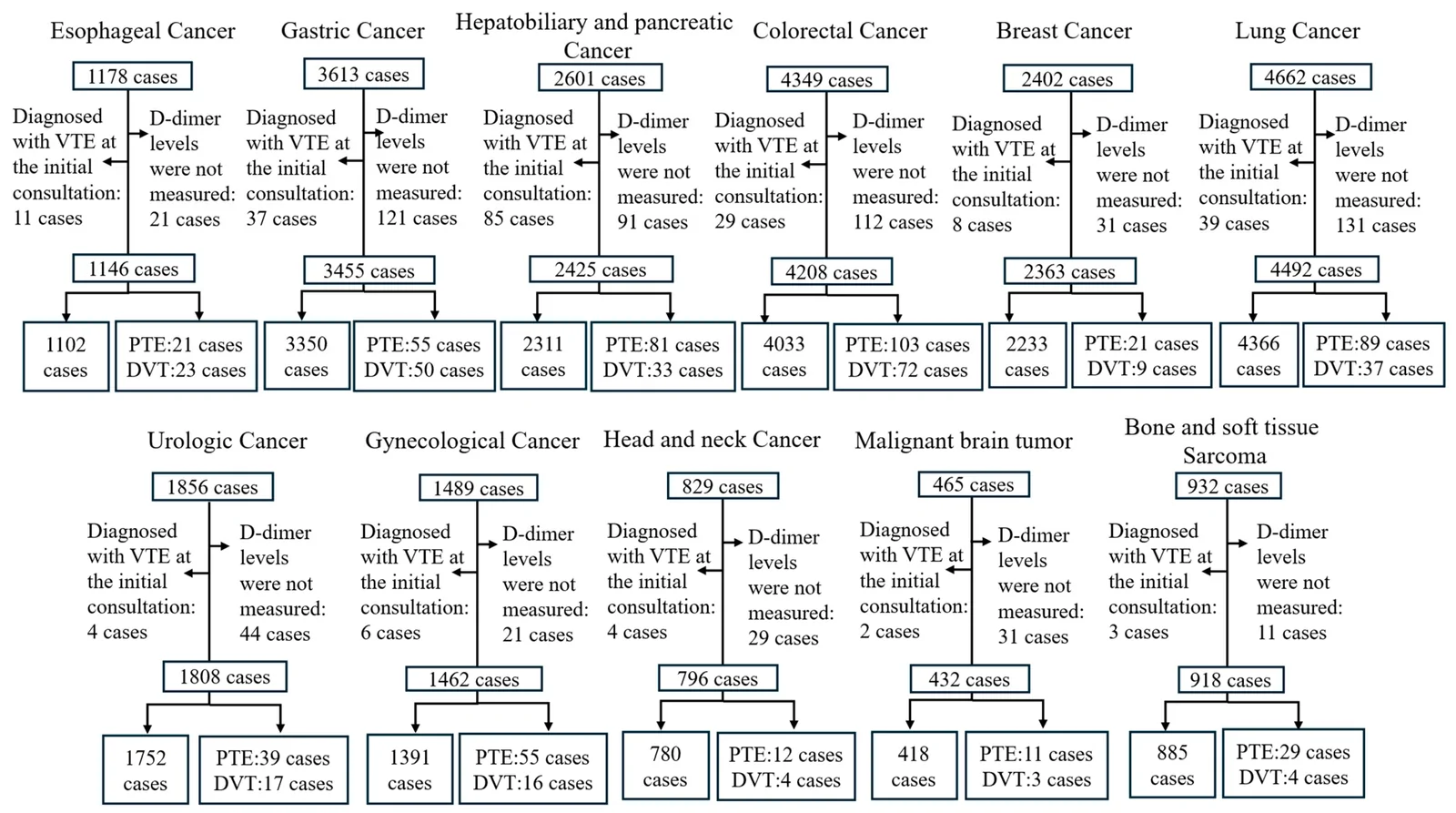
Flow diagram of patient selection and study cohort according to cancer type. DVT, deep vein thrombosis; PTE, pulmonary embolism; VTE, venous thromboembolism.

VTE occurred in 784 of 23,505 patients (3.3%) (Figure 1). The incidence of VTE varied according to cancer type, ranging from 1.3% in breast cancer to 4.9% in gynecologic malignancies.

In the univariable logistic regression analysis, ln D-dimer (OR 1.820, 95% CI 1.732–1.913; *p* < 0.001), platelet count (OR 1.007, 95% CI 1.003–1.011; *p* < 0.001), diabetes mellitus (OR 1.288, 95% CI 1.058–1.569; *p* = 0.012), and distant metastasis (OR 1.406, 95% CI 1.214–1.630; *p* < 0.001) were significantly associated with higher odds of subsequent VTE, whereas hypertension was associated with lower odds (OR 0.860, 95% CI 0.740–0.999; *p* = 0.048). Cancer type, modeled as a single categorical variable with esophageal cancer as the reference category, was significantly associated with subsequent VTE overall (Wald χ^2^ = 70.426, degrees of freedom (df) = 10; *p* < 0.001). Compared with esophageal cancer, breast cancer (OR 0.322, 95% CI 0.201–0.515; *p* < 0.001) and head and neck cancer (OR 0.514, 95% CI 0.288–0.917; *p* = 0.024) were significantly associated with lower odds of subsequent VTE in the univariable analysis (Table 3).

**Figure 2:**
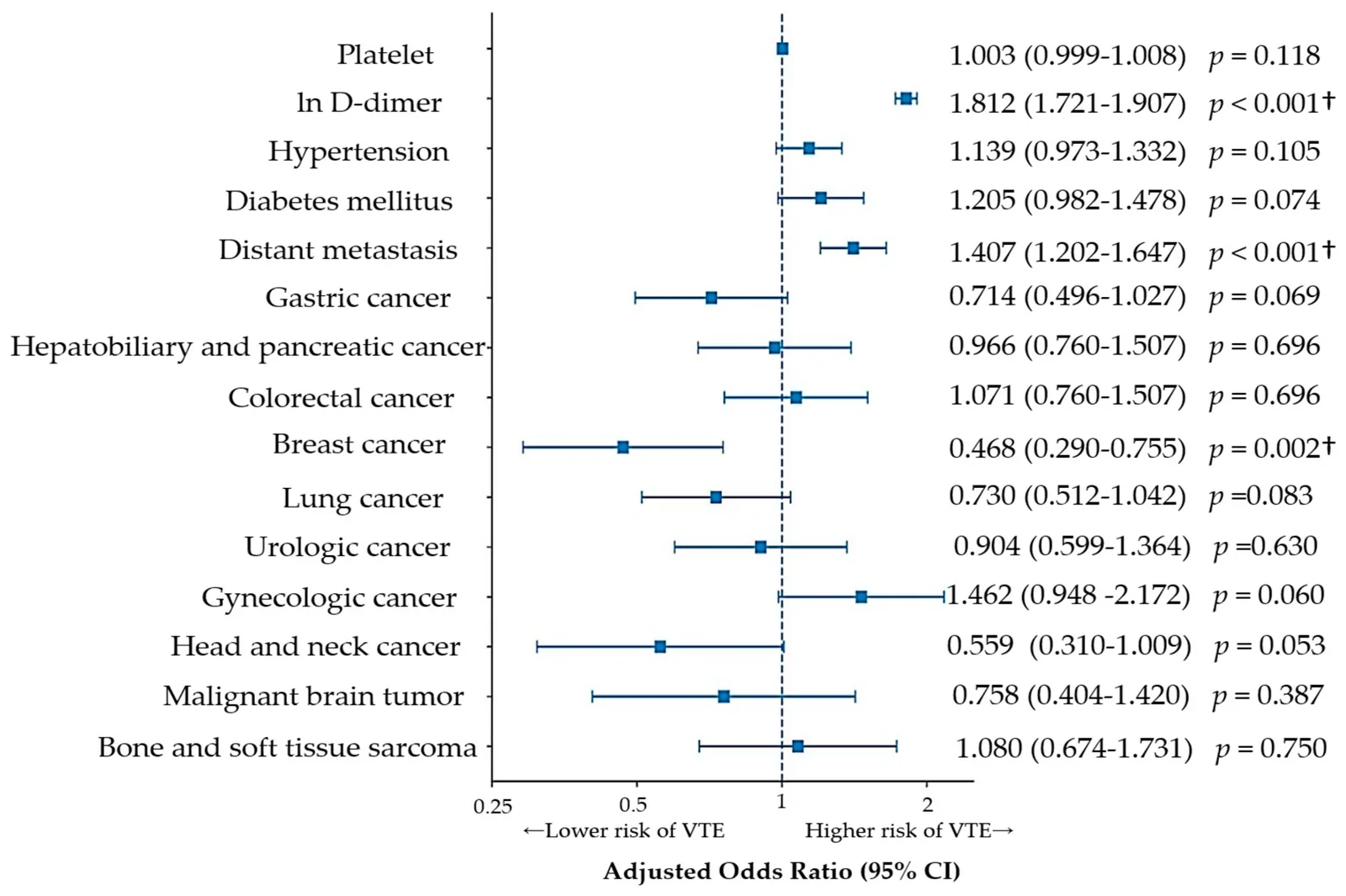
Forest plot of independent predictors of subsequent VTE. Cancer-type estimates are relative to esophageal cancer (reference). Abbreviations: VTE, venous thromboembolism; CI, confidence interval. † indicates statistically significant result (*p*-value < 0.05).

In the multivariable logistic regression analysis, ln D-dimer and distant metastasis remained independently associated with subsequent VTE. Higher ln D-dimer was strongly associated with subsequent VTE (OR 1.812, 95% CI 1.721–1.907; *p* < 0.001), and distant metastasis was also independently associated with higher odds of subsequent VTE (OR 1.407, 95% CI 1.202–1.647; *p* < 0.001). Cancer type remained significantly associated with subsequent VTE overall (Wald χ^2^ = 48.720, df = 10; *p* < 0.001). Compared with esophageal cancer, breast cancer was associated with lower odds of subsequent VTE (OR 0.468, 95% CI 0.290–0.755; *p* = 0.002), whereas no other individual cancer type showed a statistically significant association after multivariable adjustment. Platelet count, hypertension, and diabetes mellitus were not independently associated with subsequent VTE in the multivariable model (all *p* > 0.05) (Figure 2).

**Figure 3:**
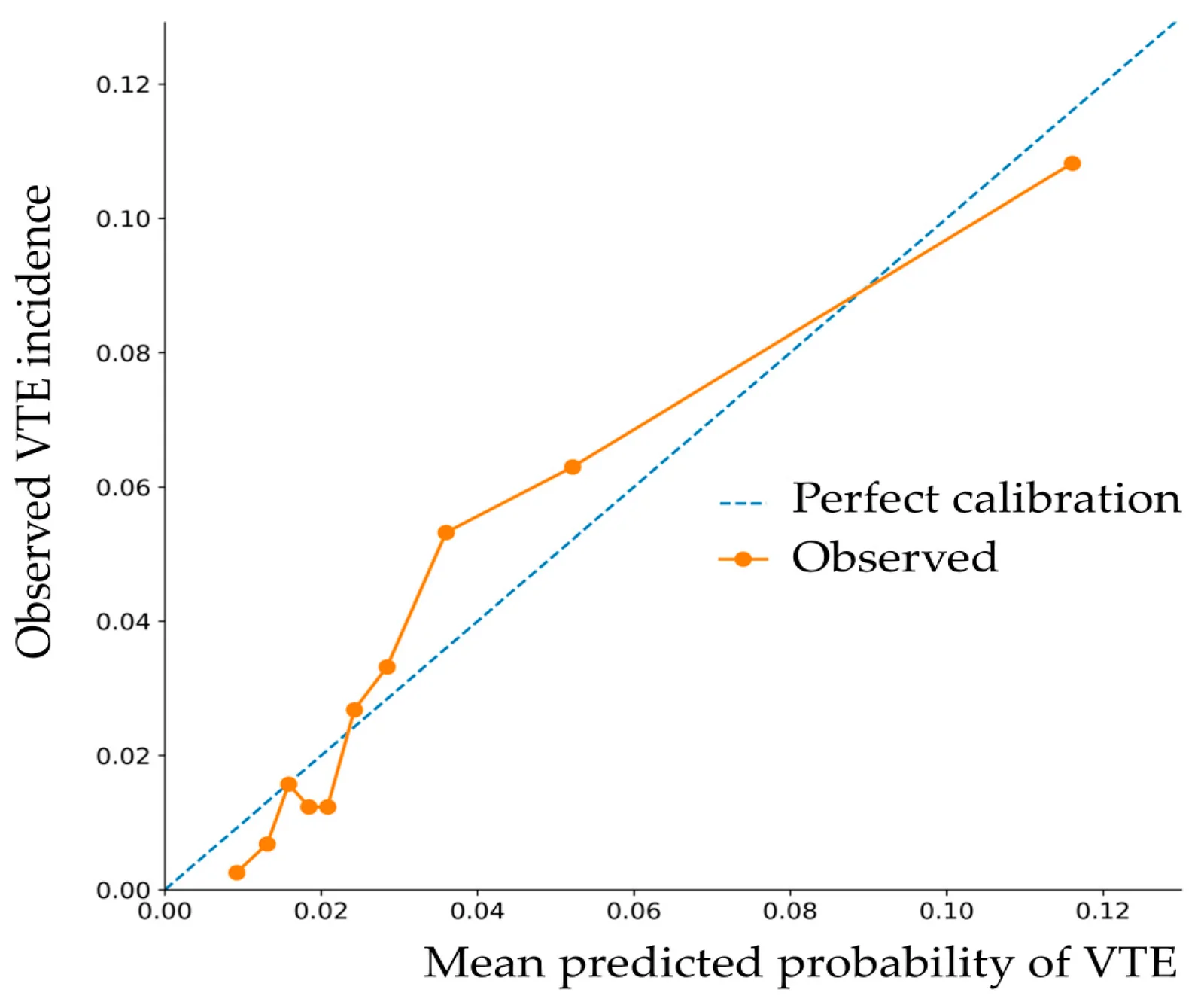
Calibration plot of the multivariable logistic regression model for subsequent VTE. Patients were divided into deciles according to the predicted probability of subsequent VTE. Mean predicted VTE probabilities were plotted against the observed VTE incidence in each decile. The dashed diagonal line represents perfect calibration.

The multivariable analysis was based on 23,495 complete cases (99.96% of the study population). Multicollinearity was assessed using VIF, which ranged from 1.025 to 4.039, with all values below 5. The model showed moderate discrimination, with an AUC) of 0.753 (95% CI 0.737–0.769). The Hosmer–Lemeshow goodness-of-fit test was statistically significant (χ^2^ = 61.269, df = 8; *p* < 0.001), and the decile-based calibration assessment showed some deviation between predicted and observed VTE risks, indicating imperfect calibration (Figure 3).

**Figure 4:**
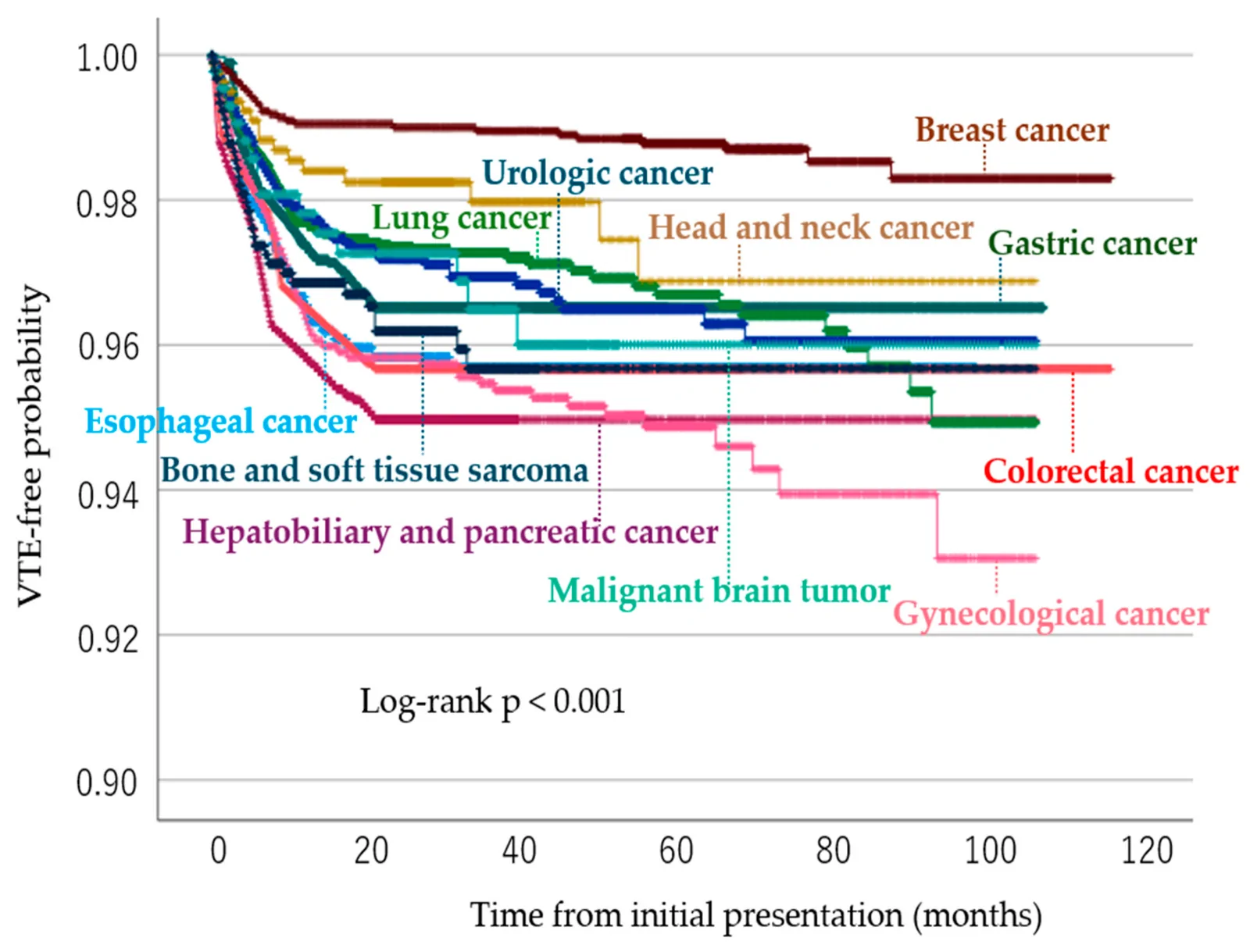
Kaplan–Meier curves for time to subsequent venous thromboembolism according to cancer type. Kaplan–Meier curves show VTE-free probability according to cancer type. Patients who did not develop VTE were censored at the end of follow-up. VTE-free survival differed significantly among the 11 cancer types (log-rank χ^2^ = 77.303, df = 10, *p* < 0.001). Abbreviations: VTE, venous thromboembolism.

In an additional time-to-event analysis accounting for differences in follow-up duration, the multivariable Cox regression analysis yielded findings consistent with those of the logistic regression analysis. Higher ln D-dimer (HR 1.737, 95% CI 1.659–1.819; *p* < 0.001) and distant metastasis (HR 1.356, 95% CI 1.166–1.576; *p* < 0.001) remained significantly associated with subsequent VTE. Cancer type was significantly associated with time to subsequent VTE overall (*p* < 0.001), with breast cancer showing a lower hazard than esophageal cancer (HR 0.407, 95% CI 0.255–0.650; *p* < 0.001). Kaplan–Meier analysis also demonstrated significant differences among cancer types (log-rank *p* < 0.001) (Figure 4). The significant interaction between ln D-dimer and ln(time) (*p* < 0.001) suggested that the association between baseline D-dimer and subsequent VTE attenuated over time.

### 3.3. Cancer-Specific Predictive Performance of D-Dimer

**Figure 5:**
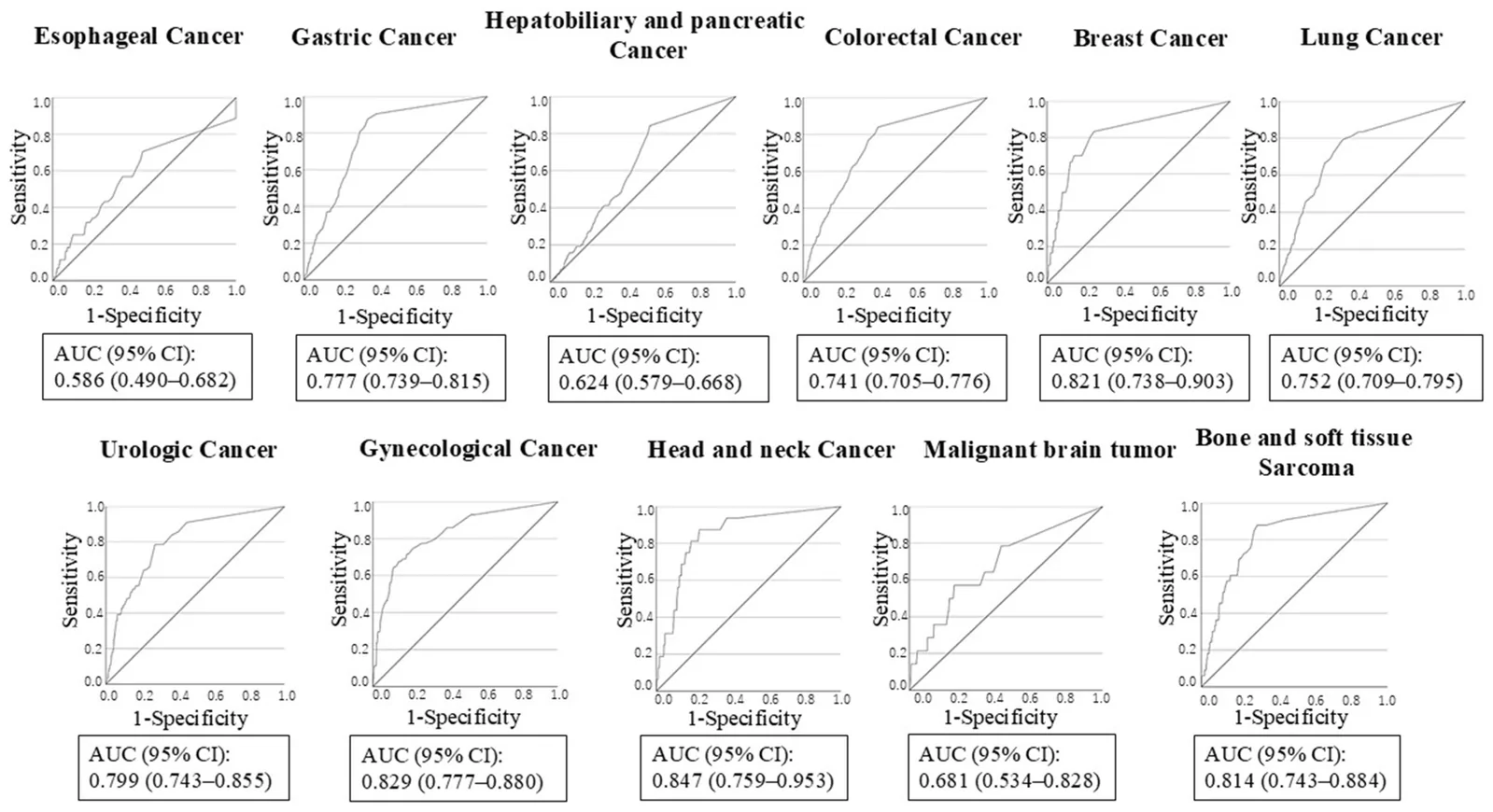
Receiver operating characteristic curves of baseline D-dimer levels for predicting subsequent venous thromboembolism in 11 cancer types. AUC, area under the curve; CI, confidence interval.

ROC analyses were subsequently performed to evaluate the predictive performance of baseline D-dimer levels according to cancer type (Table 4, Figure 5). Discriminative performances were head and neck cancer (AUC 0.847, 95% CI, 0.759–0.953), followed by gynecologic cancer (AUC 0.829, 95% CI 0.777–0.880), breast cancer (AUC 0.821, 95% CI 0.738–0.903), and bone and soft tissue sarcoma (AUC 0.814, 95% CI 0.743–0.884).

In contrast, baseline D-dimer levels showed limited discriminative ability for subsequent VTE in patients with esophageal cancer (AUC 0.586, 95% CI 0.490–0.682); the ROC analysis did not reach statistical significance (*p* = 0.053). Therefore, no optimal cutoff value was reported for esophageal cancer. Among cancer types with statistically significant ROC results, optimal D-dimer cutoff values varied considerably among cancers, ranging from 0.55 μg/mL in hepatobiliary and pancreatic cancer, colorectal cancer, and breast cancer to 2.35 μg/mL in gynecologic cancer and malignant brain tumor. Sensitivity ranged from 57.1% to 87.9%, while specificity ranged from 46.3% to 86.2%. Positive predictive values (PPVs) were relatively low, whereas negative predictive values (NPVs) were consistently high across cancer types. Bootstrap analyses using 1000 resamples demonstrated variability in the precision of the estimated cutoff values across cancer types, with wider confidence intervals observed particularly in cancer types with fewer VTE events. Internal validation using bootstrap optimism correction showed generally modest optimism in the Youden index, although greater optimism was observed for head and neck cancer and malignant brain tumor (Table 5). These findings indicate that the cancer-specific cutoff values should be interpreted as exploratory thresholds requiring external validation.

### 3.4. Factors Associated with Elevated Baseline D-Dimer Levels

Additional exploratory analyses were performed to better characterize the factors associated with baseline D-dimer elevation. In the univariable analyses, several clinical and laboratory variables were associated with elevated D-dimer levels. However, in the multivariable linear regression analysis, older age and higher platelet count were independently associated with elevated D-dimer levels (age: B = 0.007, 95% CI 0.006–0.008, *p* < 0.001; platelet count: B = 0.003, 95% CI 0.002–0.004, *p* < 0.001), whereas higher total cholesterol levels were inversely associated with D-dimer levels (B = −0.003, 95% CI −0.003 to −0.002, *p* < 0.001) (Table 6).

## 4. Discussion

To our knowledge, this is one of the largest studies to evaluate the cancer-specific predictive performance and optimal cutoff values of baseline D-dimer levels across multiple malignancies using a unified analytical approach. In this large retrospective cohort study involving patients with one of 11 different malignancies, baseline D-dimer was identified as an independent predictor of subsequent VTE development. Several risk assessment models have been developed to identify patients with cancer at increased risk of VTE, among which the Khorana score is the most widely validated model [25]. Importantly, baseline D-dimer remained independently associated with subsequent VTE after adjustment for the clinical covariates included in the present analysis, supporting its potential role as a biomarker of thrombotic risk during the subsequent course of cancer treatment. In a prospective study of 9020 cases of colorectal, lung, gastric, pancreatic, breast, and gynecologic cancers with a short follow-up period of 1 year, D-dimer abnormalities were associated with an increased risk of symptomatic VTE [26]. Our findings are consistent with these results and extend them to a cohort of over 20,000 patients that included esophageal, urologic, head and neck, brain, and bone and soft tissue tumors.

Furthermore, ROC analyses demonstrated substantial heterogeneity in the predictive performance of D-dimer levels across cancer types. Although elevated D-dimer levels have been consistently associated with cancer-associated thrombosis and poor prognosis in previous studies [8,18,20,21], few have systematically evaluated the predictive performance of baseline D-dimer levels for future VTE according to cancer type. Our findings suggest that the clinical utility of D-dimer as a risk stratification marker is not uniform across malignancies and may depend on tumor-specific biological characteristics.

Cancer-associated thrombosis is a multifactorial process involving tumor-related hypercoagulability, systemic inflammation, endothelial injury, platelet activation, and treatment-related factors [27]. Among the available biomarkers, D-dimer reflects the ongoing activation of coagulation and fibrinolysis, and it has been widely recognized as a useful marker associated with thrombotic risk in patients with cancer [8,18,20,21]. Tumor cells promote coagulation through tissue factor expression, procoagulant microparticle release, platelet activation, and inflammatory cytokine induction [11,17,28]. These mechanisms lead to thrombin generation and fibrin turnover, resulting in elevated circulating D-dimer levels.

Another important finding of this study was the independent association between distant metastasis and subsequent development of VTE. Previous studies have consistently demonstrated that advanced-stage malignancies carry a substantially higher risk of thromboembolic events [29,30,31,32]. Metastatic tumors are associated with a larger tumor burden, increased systemic inflammation, enhanced expression of procoagulant factors, and greater exposure to anticancer therapies, all of which may contribute to thrombogenesis. Therefore, patients with elevated D-dimer levels and distant metastases may represent a high-risk subgroup requiring careful surveillance for VTE.

Cancer type was significantly associated with subsequent VTE overall when modeled as a single categorical variable with esophageal cancer as the reference category. After multivariable adjustment, breast cancer was associated with lower odds of subsequent VTE compared with esophageal cancer, whereas no other individual cancer type showed a statistically significant association relative to the reference category. These findings suggest heterogeneity in VTE risk across malignancies; however, individual cancer-type estimates should be interpreted in relation to the reference category rather than as an absolute ranking of thrombotic risk. Differences in tumor biology, disease burden, treatment exposure, and patient characteristics may contribute to this heterogeneity. Although hepatobiliary and pancreatic cancer was not significantly associated with subsequent VTE compared with esophageal cancer after multivariable adjustment, this study examined the development of VTE during the course of cancer treatment and therefore excluded patients in whom VTE was already present at the initial consultation. In our cohort, patients with hepatobiliary and pancreatic cancer had the highest proportion of VTE already present at the initial consultation.

A major finding of the present study was the marked variation in the discriminative ability of D-dimer levels among cancer types. Moderate to good discriminative performance was observed across several cancer types, including head and neck cancer (AUC 0.847), gynecologic cancer (AUC 0.829), breast cancer (AUC 0.821), bone and soft tissue sarcoma (AUC 0.814), and urologic malignancies (AUC 0.799), although the confidence intervals were relatively wide for cancer types with a limited number of VTE events. In contrast, D-dimer levels demonstrated limited predictive performance in esophageal cancer (AUC 0.586), for which the ROC analysis did not reach statistical significance, and only modest performance in hepatobiliary and pancreatic malignancies (AUC 0.624). These findings highlight substantial heterogeneity in the predictive performance of baseline D-dimer across cancer types.

The limited predictive performance observed in esophageal cancer may reflect the multifactorial nature of elevated D-dimer levels in this population. Patients with esophageal cancer are frequently older adults and often present with malnutrition, chronic inflammation, and treatment-related physiological stress. These factors may elevate D-dimer levels independent of thrombus formation, thereby increasing the overlap between patients who subsequently develop VTE and those who do not. Furthermore, background factors such as smoking, alcohol consumption, living alone, and dehydration owing to dysphagia in patients with esophageal cancer may significantly contribute to thrombosis risk and potentially reduce the relative contribution of baseline D-dimer levels [2,33].

Conversely, gynecologic malignancies demonstrated good discriminative predictive performance. Previous studies have reported strong associations between tissue factor expression, hypercoagulability, and thromboembolic complications in gynecologic cancers, particularly ovarian cancer [34]. Similarly, tissue factor expression is associated with disease progression and activation of coagulation pathways in breast cancer [35]. In these malignancies, elevated D-dimer levels may more directly reflect the clinically relevant activation of the coagulation system, resulting in superior discrimination of the future risk of VTE.

Notably, head and neck cancer demonstrated the highest AUC among all cancer types. Although head and neck malignancies are not traditionally regarded as being among the highest-risk cancers for VTE, the present findings suggest that baseline D-dimer may provide useful information for VTE risk stratification in this population. However, the number of VTE events in this subgroup was limited, and bootstrap analysis demonstrated substantial uncertainty in the estimated optimal cutoff. Furthermore, bootstrap internal validation showed greater optimism in the Youden index than in several of the larger cancer subgroups. Therefore, the apparently high discriminative performance observed in head and neck cancer should be interpreted cautiously and requires validation in an independent cohort.

The optimal D-dimer cutoff values also differed substantially among cancer types, ranging from 0.55 to 2.35 μg/mL among malignancies with statistically significant ROC results. This variation suggests that a single universal D-dimer threshold may not adequately capture the heterogeneity in VTE risk across different malignancies and may reflect differences in baseline coagulation activity, inflammatory burden, tumor biology, disease stage distribution, and patient characteristics [4,6,8,10,25]. However, the present cutoff values should be interpreted as exploratory rather than definitive clinical thresholds. Bootstrap analysis demonstrated substantial variation in the precision of the estimated cutoffs, with particularly wide confidence intervals in cancer types with relatively few VTE events. Internal validation using bootstrap optimism correction similarly showed greater optimism in these smaller subgroups, supporting the need for external validation before these cancer-specific thresholds can be applied clinically.

Another notable finding was that PPVs were relatively low, whereas NPVs were consistently high across cancer types. This pattern is partly attributable to the low incidence of subsequent VTE in the study population and therefore should not be interpreted as evidence that D-dimer alone can reliably rule out future VTE. Rather, baseline D-dimer may provide additional information for identifying patients at relatively low subsequent VTE risk and may be most useful when incorporated into a multifactorial risk assessment together with cancer type, disease extent, and other clinical factors.

Notably, although baseline ln-transformed D-dimer was significantly associated with subsequent VTE in the multivariable Cox model (HR 1.737, 95% CI 1.659–1.819; *p* < 0.001), its predictive association diminished over time following the initial measurement. This temporal attenuation was supported by the statistically significant interaction between ln-transformed D-dimer and ln (time) (HR 0.652, 95% CI 0.623–0.683; *p* < 0.001). Given that the majority of VTE events in the Kaplan–Meier analysis occurred within approximately 2 years of the start of follow-up, these findings should be interpreted within the context of the temporal distribution of VTE events in our cohort. Consequently, baseline D-dimer levels may primarily reflect the thrombogenic potential present at or near the time of cancer diagnosis, rather than an unchanging, long-term susceptibility to VTE. As the clinical course progresses, changes in tumor burden, anticancer treatments, surgery, disease progression, and other patient-related factors can significantly alter thrombogenic risk; this likely diminishes the prognostic value derived from a single baseline measurement. These findings suggest that baseline D-dimer levels are particularly useful for identifying patients at high risk of VTE during the relatively early stages following a cancer diagnosis. Conversely, for long-term risk assessment, re-evaluating D-dimer levels and other dynamic risk factors may yield more informative data.

Additional exploratory analyses were performed to identify determinants of baseline D-dimer levels. Previous studies have reported associations between D-dimer levels and advanced age, inflammatory conditions, liver dysfunction, renal dysfunction, and autoimmune disorders [36,37,38]. Although infection, hepatic dysfunction, and renal dysfunction contribute to elevated D-dimer levels, none of these factors was significantly associated with D-dimer levels in our cohort. This discrepancy may reflect the predominant influence of cancer-related hypercoagulability and systemic inflammation in patients with malignancy, which may outweigh the effects of concomitant infections or organ dysfunction. Interestingly, advanced age, platelet count, and lower serum cholesterol levels remained independently associated with elevated D-dimer levels in our multivariable analysis.

There are several possible explanations for the discrepancy. First, the exceptionally large sample size of the present study enabled simultaneous adjustment for multiple clinical variables, thereby reducing the influence of confounding factors. Second, as the study population exclusively comprised patients with malignancies, cancer-related hypercoagulability may have outweighed the effects of comorbidity-related coagulation activation. Third, serum cholesterol levels and platelet counts may better reflect the integrated effects of cancer burden, nutritional status, and coagulation pathway activation than individual clinical characteristics alone. Hypocholesterolemia is a component of the CONUT score and has been linked to poor nutritional status and an unfavorable prognosis in patients with cancer. Therefore, low cholesterol levels may serve as surrogate markers of advanced disease burden and cancer-related systemic inflammation, both of which may contribute to the activation of the coagulation system [39]. Platelet activation plays a central role in cancer-associated thrombosis and has been incorporated into established risk prediction models such as the Khorana score [21]. Furthermore, elevated platelet-related inflammatory markers, including the platelet-to-lymphocyte ratio, are associated with adverse oncologic outcomes in multiple malignancies [40]. Low cholesterol levels and thrombocytosis may reflect increased thrombus formation caused by tumor progression. In fact, the presence of distant metastases was a significant risk factor for subsequent VTE formation.

Our data showed that D-dimer levels increased with age, and higher D-dimer levels were associated with a higher risk of developing VTE. However, VTE did not occur more frequently in older patients with cancer. As the optimal ROC-derived cutoff differed among cancer types, the observed association between age and D-dimer levels further supports the idea that a uniform D-dimer threshold may not be appropriate across diverse cancer patient populations, particularly in older patients.

The present findings suggest that, within a large oncologic population, baseline D-dimer levels may be more strongly influenced by biological processes associated with cancer-related hypercoagulability than by demographic characteristics or comorbid conditions. Importantly, despite the association of platelet count and cholesterol levels with D-dimer elevation, only D-dimer remained an independent predictor of subsequent VTE development, indicating that D-dimer may function as a composite biomarker that integrates multiple prothrombotic mechanisms.

Early identification of patients at high risk of VTE may facilitate closer surveillance and appropriate thromboprophylactic strategies because cancer-associated VTE is associated with substantial morbidity and mortality [5,41]. Recent studies have identified growth differentiation factor-15 and high-sensitivity troponin T as risk factors for VTE in patients with cancer; however, neither biomarker is readily measured in routine clinical practice [42]. The identification of high-risk patients using readily available biomarkers such as D-dimer may facilitate individualized surveillance and thromboprophylactic strategies in selected cancer populations [43].

This study had some limitations. First, this was a retrospective study conducted at a single institution, and residual confounding cannot be excluded. Several potentially important confounders were not available, including detailed cancer stage, treatment-related factors, performance status, central venous catheter use, and baseline antithrombotic therapy or thromboprophylaxis. These unmeasured factors may have influenced the observed associations between baseline D-dimer and subsequent VTE, and residual confounding cannot be excluded. Therefore, the findings should be interpreted as associative rather than causal. Second, VTE screening and imaging evaluations were performed according to routine clinical practice rather than a predefined protocol, potentially introducing detection bias. PTE included both symptomatic events and incidental events detected on CT performed for cancer staging, treatment response assessment, or other clinical indications, whereas DVT was restricted to symptomatic proximal lower-extremity events. Because symptoms preceding PTE diagnosis were not systematically documented in this retrospective electronic medical record–based study, symptomatic and incidental PTE could not be reliably classified and reported separately. Furthermore, patients with advanced or metastatic disease may undergo more frequent CT imaging and therefore have greater opportunities for incidental PTE detection. Accordingly, the observed association between distant metastasis and subsequent VTE may partly reflect differences in surveillance intensity rather than an exclusively biological increase in thrombotic risk. In addition, although we performed a Cox time-to-event analysis to account for differences in follow-up duration, death was not collected as a separate competing-event variable in the prespecified analytic dataset. Therefore, Fine–Gray competing-risk regression and cumulative incidence functions accounting for death could not be performed. Because mortality differs substantially among cancer types, competing mortality may have influenced the observed between-cancer differences in subsequent VTE. Furthermore, the proportional-hazard assumption was not supported for baseline D-dimer, suggesting that its association with subsequent VTE attenuated over time. Accordingly, the Cox analysis should be interpreted as a complementary time-to-event analysis, and the cancer-specific findings require validation in prospective studies incorporating mortality as a competing event. Third, some cancer-specific subgroups contained relatively small numbers of VTE events, which may have affected the precision of the estimated AUC values and the optimal cutoff points. Although bootstrap analysis and optimism correction were performed for internal validation, substantial uncertainty in the estimated cutoff values remained in some smaller subgroups, particularly those with few VTE events. Therefore, these cancer-specific cutoff values should be considered exploratory rather than definitive clinical thresholds. Fourth, patients without baseline D-dimer measurements were excluded during cohort construction, and detailed clinical and laboratory variables were not extracted for these patients. Therefore, we were unable to compare the clinical characteristics of the 643 patients excluded because of absent baseline D-dimer measurements with those of the included patients. Because the decision to measure D-dimer in routine clinical practice may have been influenced by patient characteristics or clinical suspicion of thrombosis, exclusion of these patients may have introduced selection bias. Fifth, although the multivariable model showed moderate discrimination, calibration was not perfect, as indicated by the significant Hosmer–Lemeshow test and deviations between predicted and observed risks in the decile-based calibration analysis. Therefore, the model should be regarded primarily as an explanatory model for identifying factors associated with subsequent VTE rather than as a fully validated clinical prediction model.

Finally, although bootstrap-based internal validation was performed, external validation was not conducted. Therefore, the cancer-specific cutoff values identified in this study require validation in independent cohorts before they can be considered for routine clinical application.

## 5. Conclusions

Baseline D-dimer level was an independent predictor of subsequent VTE development in patients with cancer. The predictive performance and exploratory D-dimer thresholds varied across cancer types, suggesting that the clinical interpretation of D-dimer may need to account for cancer type. Prospective studies with external validation are warranted to confirm these findings and determine their potential clinical utility.

## Figures and Tables

**Table 1:** Baseline characteristics of patients with one of 11 different malignancies.

	EC	GC	HBP	CRC	BC	LC	UC	GNC	HNC	MBT	BSS
Number (cases)	1146	3455	2425	4208	2363	4492	1808	1462	796	432	918
Age	78 (72–85)	77 (70–87)	78 (73–87)	77 (68–85)	68 (56–78)	76 (63–86)	78 (70–86)	64 (52–77)	77 (67–84)	66 (48–80)	69 (52–81)
Sex (male/female) n (%)	917 (80.0%)/ 229 (20.0%)	2382 (68.9%)/ 1073 (31.1%)	1431 (59.0%)/ 994 (41.0%)	2268 (53.9%)/ 1940 (46.1%)	20 (0.8%)/ 2343 (99.2%)	2789 (62.1%)/ 1703 (37.9%)	1317 (72.8%)/ 491 (27.2%)	0 (0.0%)/ 1462 (100.0%)	625 (78.5%)/ 171 (21.5%)	221 (51.2%)/ 211 (48.8%)	500 (54.5%)/ 418 (45.5%)
White blood cell count	5965 (4460–7630)	5260 (4800–7440)	5820 (4550–7590)	6270 (5080–7770)	5550 (4430–7390)	6040 (5090–7650)	6100 (4880–7540)	5480 (4900–7440)	5950 (4620–7750)	6040 (4740–7700)	6160 (4740–7670)
Hemoglobin	11.0 (9.4–13.0)	12.3 (10.9–14.2)	11.3 (9.0–14.1)	12.1 (10.1–14.3)	12.1 (11.0–14.2)	13.6 (11.1–14.8)	12.2 (9.8–14.3)	11.2 (9.7–14.0)	12.1 (9.8–14.3)	14.3 (13.1–15.3)	13.5 (11.4–15.0)
Platelet count (×10^4^)	24.0 (17.3–32.1)	21.2 (14.1–27.8)	28.7 (17.5–32.9)	24.1 (17.1–27.1)	18.9 (15.7–25.4)	21.9 (15.7–25.4)	26.9 (17.2–34.0)	21.7 (17.3–25.2)	20.4 (16.4–29.3)	22.1 (14.5–30.7)	27.3 (20.7–34.1)
BMI	19.9 (17.7–22.5)	20.7 (18.1–23.3)	21.1 (18.8–23.8)	22.0 (19.7–24.3)	22.2 (19.9–24.9)	21.5 (19.2–23.9)	22.5 (19.9–24.9)	21.5 (18.9–24.9)	20.5 (18.1–22.9)	20.6 (18.4–23.8)	21.9 (19.4–24.4)
D-dimer (μg/mL)	0.5 (0.5–1.6)	0.5 (0.5–1.6)	0.7 (0.5–2.4)	0.5 (0.5–1.5)	0.5 (0.5–0.6)	0.5 (0.5–1.6)	0.5 (0.5–1.3)	0.7 (0.7–1.5)	0.5 (0.5–1.7)	0.6 (0.6–2.1)	0.5 (0.5–1.3)
Total protein (g/dL)	7.0 (6.6–7.3)	6.9 (6.5–7.2)	7.0 (6.6–7.4)	7.0 (6.7–7.3)	7.2 (6.8–7.5)	7.1 (6.7–7.4)	7.2 (6.7–7.4)	7.1 (6.7–7.4)	7.0 (6.8–7.5)	7.0 (6.6–7.3)	7.1 (6.8–7.4)
Albumin (g/dL)	4.1 (3.8–4.4)	4.2 (3.8–4.4)	4.0 (3.6–4.4)	4.1 (3.5–4.4)	4.2 (3.1–4.5)	4.2 (3.8–4.4)	4.2 (4.0–4.5)	4.3 (4.0–4.5)	4.2 (3.9–4.5)	4.3 (4.0–4.5)	4.3 (4.0–4.5)
Total cholesterol (mg/dL)	187 (160–215)	185 (160–211)	176 (149–205)	194 (169–220)	204 (180–229)	188 (162–214)	187 (161–204)	196 (173–221)	189 (162–212)	188 (156–209)	186 (162–215)
Triglycerides (mg/dL)	98 (71–134)	96 (70–136)	97 (73–136)	104 (74–146)	103 (72–147)	110 (80–153)	117 (83–167)	99 (69–145)	99 (74–145)	94 (64–137)	110 (77–156)
CRP (mg/dL)	0.3 (0.2–0.6)	0.5 (0.2–0.7)	0.4 (0.2–0.6)	0.6 (0.2–0.8)	0.3 (0.1–0.6)	0.4 (0.1–0.6)	0.5 (0.2–0.9)	0.4 (0.2–0.6)	0.4 (0.2–0.7)	0.2 (0.1–0.3)	0.4 (0.2–0.6)
Hypertension, n (%)	401 (35.0)	1001 (29.0)	1019 (42.0)	1557 (37.0)	587 (24.8)	1314 (29.3)	353 (19.5)	197 (13.5)	174 (21.9)	112 (25.9)	132 (14.4)
Diabetes, n (%)	216 (18.8)	549 (15.9)	553 (22.8)	667 (15.9)	76 (3.2)	442 (9.8)	180 (10.0)	55 (3.8)	106 (13.3)	47 (10.9)	75 (8.2)
Current smoking, n (%)	88 (7.7)	163 (4.7)	41 (1.7)	131 (3.1)	17 (0.7)	229 (5.1)	27 (1.5)	16 (1.1)	55 (6.9)	6 (1.4)	19 (2.1)
Hepatic dysfunction, n (%)	5 (0.4)	44 (1.3)	55 (2.3)	9 (0.2)	21 (0.9)	21 (0.5)	5 (0.3)	11 (0.8)	2 (0.3)	3 (0.7)	36 (3.9)
Renal dysfunction, n (%)	24 (2.1)	78 (2.3)	48 (2.0)	93 (2.2)	51 (2.2)	98 (2.2)	36 (2.0)	31 (2.1)	18 (2.3)	13 (3.0)	18 (2.0)
Autoimmune disease, n (%)	20 (1.7)	62 (1.8)	52 (2.1)	74 (1.8)	41 (1.7)	81 (1.8)	29 (1.6)	28 (1.9)	13 (1.6)	9 (2.1)	14 (1.5)
Infection (+), n (%)	18 (1.6)	54 (1.6)	37 (1.5)	64 (1.5)	37 (1.6)	69 (1.5)	28 (1.5)	23 (1.6)	12 (1.5)	7 (1.6)	14 (1.5)
Distant metastasis, n (%)	417 (36.4)	893 (25.8)	1122 (46.4)	765 (18.2)	169 (7.2)	1284 (28.6)	415 (23.0)	198 (13.5)	77 (9.7)	19 (4.4)	76 (8.3)

Data are presented as medians (interquartile ranges) for continuous variables and as numbers (percentages) for categorical variables. Abbreviations: EC, esophageal cancer; GC, gastric cancer; HBP, hepatobiliary and pancreatic cancer; CRC, colorectal cancer; BC, breast cancer, LC, lung cancer; UC, urologic cancer; GNC, gynecological cancer; HNC, head and neck cancer; MBT, malignant brain tumor; BSS, bone and soft tissue sarcoma; BMI, body mass index; CRP, C-reactive protein.

**Table 2:** Baseline characteristics according to subsequent venous thromboembolism status.

Variable	Non-VTE (n = 22,721)	VTE (n = 784)	*p*-Value
Age	77 (67–85)	77 (69–84)	0.544
Male sex, n (%)	12,065 (53.1)	410 (52.3)	0.657
Cancer type, n (%)			<0.001 †
Esophageal	1102 (4.9)	44 (5.6)	
Gastric cancer	3350 (14.7)	105 (13.4)	
HBP cancer	2311 (10.2)	114 (14.5)	
Colorectal cancer	4033 (17.8)	175 (22.3)	
Breast cancer	2333 (10.3)	30 (3.8)	
Lung cancer	4366 (19.2)	126 (16.1)	
Urologic cancer	1752 (7.7)	56 (7.1)	
Gynecologic cancer	1391 (6.1)	71 (9.1)	
Head and neck cancer	780 (3.4)	16 (2.0)	
Malignant brain tumor	418 (1.8)	14 (1.8)	
Bone and soft tissue sarcoma	885 (3.9)	33 (4.2)	
White blood cell count	5.84 (4.80–7.59)	6.22 (4.95–7.80)	0.003 †
Hemoglobin	12.4 (10.4–14.3)	12.1 (10.0–14.4)	0.316
Platelet count (×10^4^)	14.9 (6.4–26.5)	19.8 (7.0–29.2)	<0.001 †
BMI	21.5 (19.1–24.0)	21.2 (18.7–24.0)	0.083
D-dimer (μg/mL)	0.5 (0.5–1.4)	2.0 (0.9–5.7)	<0.001 †
Total protein (g/dL)	7.0 (6.7–7.4)	7.0 (6.6–7.4)	0.502
Albumin (g/dL)	4.2 (3.8–4.4)	4.1 (3.7–4.4)	0.014 †
Total cholesterol (mg/dL)	190 (164–216)	181 (164–215)	0.041 †
Triglycerides (mg/dL)	108 (74–147)	103 (78–148)	0.016 †
CRP (mg/dL)	0.4 (0.2–0.6)	0.4 (0.2–0.6)	0.485
Hypertension, n (%)	7068 (31.1)	270 (34.4)	0.048 †
Diabetes mellitus, n (%)	2844 (12.5)	122 (15.6)	0.012 †
Current smoking, n (%)	720 (3.2)	33 (4.2)	0.104
Hepatic dysfunction, n (%)	201 (0.9)	11 (1.4)	0.131
Renal dysfunction, n (%)	490 (2.2)	18 (2.3)	0.792
Autoimmune disease, n (%)	407 (1.8)	16 (2.0)	0.605
Infection, n (%)	352 (1.5)	11 (1.4)	0.744
Distant metastasis, n (%)	6799 (29.9)	294 (37.5)	<0.001 †

Data are presented as median (interquartile range) or n (%). Continuous variables were compared using the Mann–Whitney U test, and categorical variables were compared using Pearson’s chi-square test. BMI, body mass index; CRP, C-reactive protein; VTE, venous thromboembolism. HBP, hepatobiliary and pancreatic. † indicates statistically significant result (*p*-value < 0.05).

**Table 3:** Univariable and multivariable logistic regression analysis for subsequent VTE.

Variable	Univariable OR (95% CI)	*p*-Value	Multivariable OR (95% CI)	*p*-Value
Age	1.001 (0.996–1.006)	0.770	—	—
Sex	1.033 (0.896–1.191)	0.657	—	—
WBC	1.017 (1.000–1.036)	0.056	—	—
Hb	0.986 (0.959–1.014)	0.332	—	—
PLT	1.007 (1.003–1.011)	<0.001 †	1.003 (0.999–1.008)	0.118
BMI	1.000 (1.000–1.000)	0.744	—	—
TP	0.990 (0.939–1.044)	0.713	—	—
Albumin	1.015 (0.946–1.089)	0.684	—	—
Total cholesterol	1.000 (0.998–1.001)	0.624	—	—
TG	1.001 (1.000–1.001)	0.154	—	—
CRP	0.956 (0.837–1.092)	0.507	—	—
ln-D-dimer	1.820 (1.732–1.913)	<0.001 †	1.812 (1.721–1.907)	<0.001 †
Hypertension	0.860 (0.740–0.999)	0.048 †	1.139 (0.973–1.332)	0.105
Diabetes mellitus	1.288 (1.058–1.569)	0.012 †	1.205 (0.982–1.478)	0.074
Smoking	1.385 (0.974–1.969)	0.069	—	—
Hepatic dysfunction	1.594 (0.865–2.938)	0.135	—	—
Renal dysfunction	1.066 (0.662–1.716)	0.792	—	—
Autoimmune disease	1.142 (0.690–1.892)	0.606	—	—
Infection	0.904 (0.494–1.655)	0.744	—	—
Distant metastasis	1.406 (1.214–1.630)	<0.001 †	1.407 (1.202–1.647)	<0.001 †
Cancer type, overall	—	<0.001 ^1^†	—	<0.001 ^2^†
Esophageal cancer	Reference	—	Reference	—
Gastric cancer	0.785 (0.549–1.123)	0.186	0.714 (0.496–1.027)	0.069
Hepatobiliary and pancreatic cancer	1.235 (0.866–1.762)	0.243	0.966 (0.670–1.392)	0.852
Colorectal cancer	1.087 (0.776–1.523)	0.629	1.071 (0.760–1.507)	0.696
Breast cancer	0.322 (0.201–0.515)	<0.001 †	0.468 (0.290–0.755)	0.002 †
Lung cancer	0.723 (0.510–1.025)	0.069	0.730 (0.512–1.042)	0.083
Urologic cancer	0.801 (0.536–1.197)	0.278	0.904 (0.599–1.364)	0.630
Gynecologic cancer	1.278 (0.871–1.877)	0.210	1.462 (0.984–2.172)	0.060
Head and neck cancer	0.514 (0.288–0.917)	0.024 †	0.559 (0.310–1.009)	0.053
Malignant brain tumor	0.839 (0.455–1.547)	0.573	0.758 (0.404–1.420)	0.387
Bone and soft tissue sarcoma	0.934 (0.590–1.479)	0.771	1.080 (0.674–1.731)	0.750

Multivariable analysis was performed using complete cases (n = 23,495). Abbreviations: WBC, white blood cell count; Hb, hemoglobin; PLT, platelet count; BMI, body mass index; TP, total protein; Alb, albumin; TG, triglycerides; CRP, C-reactive protein; ln D-dimer, natural logarithm-transformed D-dimer; VTE, venous thromboembolism; B, regression coefficients (B); SE, standard error; OR, odds ratio; CI, confidence interval. ^1^ Overall cancer-type effect in the univariable model: Wald χ^2^ = 70.426, df = 10, *p* < 0.001. ^2^ Overall cancer-type effect in the multivariable model: Wald χ^2^ = 48.720, df = 10, *p* < 0.001. † indicates statistically significant result (*p*-value < 0.05).

**Table 4:** Predictive performance of baseline D-dimer for subsequent VTE according to cancer type.

Cancer Type	AUC (95% CI)	*p*-Value	Cutoff, μg/mL (Bootstrap 95% CI)	Sensitivity, % (95% CI)	Specificity, % (95% CI)	PPV, %	NPV, %
Esophageal cancer	0.586 (0.490–0.682)	0.053	—	—	—	—	—
Gastric cancer	0.777 (0.739–0.815)	<0.001 †	0.75 (0.55–1.05)	87.6 (79.8–93.2)	65.2 (63.6–66.8)	7.3	99.4
HBP cancer	0.624 (0.579–0.668)	<0.001 †	0.55 (0.55–0.65)	84.2 (76.2–90.4)	46.3 (44.3–48.4)	7.2	98.3
Colorectal cancer	0.741 (0.705–0.776)	<0.001 †	0.55 (0.55–0.75)	84.0 (77.7–89.1)	59.4 (57.8–60.9)	8.2	98.8
Breast cancer	0.821 (0.738–0.903)	<0.001 †	0.55 (0.55–1.25)	83.3 (65.3–94.4)	74.4 (72.5–76.1)	4.0	99.7
Lung cancer	0.752 (0.709–0.795)	<0.001 †	0.95 (0.85–1.55)	79.4 (71.2–86.1)	65.7 (64.3–67.1)	6.3	99.1
Urologic cancer	0.799 (0.743–0.855)	<0.001 †	1.15 (0.55–1.45)	78.6 (65.6–88.4)	72.5 (70.4–74.6)	8.4	99.1
Gynecologic cancer	0.829 (0.777–0.880)	<0.001 †	2.35 (0.85–2.35)	67.6 (55.5–78.2)	86.2 (84.3–88.0)	12.6	98.8
Head and neck cancer	0.847 (0.759–0.953)	<0.001 †	1.75 (0.65–3.65)	87.5 (61.7–98.4)	76.7 (73.5–79.6)	7.1	99.7
Malignant brain tumor	0.681 (0.534–0.828)	0.021 †	2.35 (0.65–21.00)	57.1 (28.9–82.3)	76.8 (72.4–80.8)	7.6	98.2
Bone and soft tissue sarcoma	0.814 (0.743–0.884)	<0.001 †	0.85 (0.85–1.95)	87.9 (71.8–96.6)	70.1 (66.9–73.1)	9.9	99.4

Optimal cutoff values were determined by maximizing the Youden index. The 95% confidence intervals for the cutoff values were estimated using 1000 bootstrap resamples with the percentile method. The 95% confidence intervals for sensitivity and specificity were calculated using the Clopper–Pearson exact method. The *p*-values correspond to tests of the null hypothesis that the AUC equals 0.5. Abbreviations: AUC, area under the curve; CI, confidence interval; PPV, positive predictive value; NPV, negative predictive value; VTE, venous thromboembolism; HBP, hepatobiliary and pancreatic; † indicates statistically significant result (*p*-value < 0.05).

**Table 5:** Bootstrap Internal Validation of Cancer-Specific Youden Indices for D-Dimer Cutoff Values.

Cancer Type	Apparent Youden J	Bootstrap Optimism	Optimism-Corrected Youden J
Esophageal cancer	0.215	0.047	0.168
Gastric cancer	0.528	0.011	0.518
Hepatobiliary and pancreatic cancer	0.305	0.003	0.303
Colorectal cancer	0.434	0.006	0.428
Breast cancer	0.577	0.041	0.536
Lung cancer	0.450	0.015	0.436
Urologic cancer	0.511	0.016	0.495
Gynecologic cancer	0.538	0.027	0.511
Head and neck cancer	0.642	0.060	0.582
Malignant brain tumor	0.339	0.088	0.251
Bone and soft tissue sarcoma	0.579	0.026	0.553

Youden J = sensitivity + specificity − 1. Bootstrap optimism was estimated using 1000 bootstrap resamples. The optimism-corrected Youden J was calculated by subtracting the estimated optimism from the apparent Youden J.

**Table 6:** Univariable and multivariable linear regression analysis of factors associated with baseline D-dimer levels.

	Univariable Analysis			Multivariable Analysis		
Variable	B (95%CI)	β	*p*-Value	B (95%CI)	β	*p*-Value
Age	0.007 (0.006–0.008)	0.103	<0.001 †	0.007 (0.006–0.008)	0.096	<0.001 †
Sex	0.100 (0.074–0.126)	0.049	<0.001 †	0.025 (−0.002–0.051)	0.012	0.070
WBC	−0.003 (−0.007–0.001)	−0.008	0.195			
Hb	0.003 (−0.002–0.008)	0.008	0.214			
PLT	0.002 (0.002–0.003)	0.037	<0.001 †	0.003 (0.002–0.004)	0.051	<0.001 †
BMI	<0.001 (−0.001-<0.001)	−0.011	0.079			
TP	−0.002 (−0.004–0.001)	−0.007	0.269			
Alb	−0.017 (−0.032–−0.002)	−0.014	0.029 †	0.014 (−0.001–0.029)	0.012	0.065
Cholesterol	−0.003 (−0.003–−0.003)	−0.122	<0.001 †	−0.003 (−0.003–−0.002)	−0.113	<0.001 †
TG	<0.001 (<0.001–<0.001)	−0.014	0.026 †	<0.001 (<0.001-<0.001)	0.009	0.195
CRP	<0.001 (−0.023–0.024)	<0.001	0.974			
Hypertension	0.023 (−0.005–0.051)	0.010	0.111			
Diabetes mellitus	−0.022 (−0.061–0.017)	−0.007	0.266			
Smoking	−0.069 (−0.142–0.005)	−0.012	0.067			
Hepatic dysfunction	0.014 (−0.123–0.151)	0.001	0.840			
Renal dysfunction	−0.026 (−0.115–0.063)	−0.004	0.562			
Autoimmune disease	0.070 (−0.027–0.168)	0.009	0.158			
Infection	0.039 (−0.067–0.144)	0.005	0.471			
Distant metastasis	0.028 (0.000–0.056)	0.013	0.051			

Abbreviations: WBC, white blood cell count; Hb, hemoglobin; PLT, platelet count; BMI, body mass index; TP, total protein; Alb, albumin; TG, triglycerides; CRP, C-reactive protein; B, unstandardized coefficients (B); CI, confidence interval; β, standardized coefficients (β). † indicates statistically significant result (*p*-value < 0.05).

## Data Availability

The original contributions of this study are included in the article. Further inquiries can be directed to the corresponding author.

## Abbreviations

The following abbreviations are used in this manuscript:

VTE	Venous thromboembolism
ROC	Receiver operating characteristic
CRP	C-reactive protein
OR	Odds ratio
CI	Confidence interval
AUC	Area under the curve
WBC	White blood cell count
Hb	Hemoglobin
PLT	Platelet count
BMI	Body mass index
TP	Total protein
Alb	Albumin
TG	Triglycerides
HBP	Hepatobiliary and pancreatic
SE	Standard error
DVT	Deep vein thrombosis
PTE	Pulmonary embolism
